# Optimizing Testimonials for Behavior Change in a Digital Intervention for Binge Eating: Human-Centered Design Study

**DOI:** 10.2196/59691

**Published:** 2025-03-21

**Authors:** Isabel R Rooper, Adrian Ortega, Thomas A Massion, Tanvi Lakhtakia, Macarena Kruger, Leah M Parsons, Lindsay D Lipman, Chidiebere Azubuike, Emily Tack, Katrina T Obleada, Andrea K Graham

**Affiliations:** 1 Center for Behavioral Intervention Technologies, Feinberg School of Medicine, Northwestern University, 750 N Lake Shore Dr, 10th Floor, Chicago, IL, 60611, United States, 1 3125035266; 2 Medical Social Sciences, Feinberg School of Medicine, Northwestern University, Chicago, IL, United States; 3 Preventive Medicine, Feinberg School of Medicine, Northwestern University, Chicago, IL, United States; 4 Department of Psychiatry and Behavioral Sciences, Feinberg School of Medicine, Northwestern University, Chicago, IL, United States; 5 Potocsnak Family Division of Adolescent and Young Adult Medicine, Ann & Robert H. Lurie Children’s Hospital of Chicago, Lurie Children's Hospital, Chicago, IL, United States

**Keywords:** health behavior, health narratives, binge eating, user engagement, personalization, behavior change, digital health, intervention, human-centered design, behavioral health, preferences

## Abstract

**Background:**

Testimonials from credible sources are an evidence-based strategy for behavior change. Behavioral health interventions have used testimonials to promote health behaviors (eg, physical activity and healthy eating). Integrating testimonials into eating disorder (ED) interventions poses a nuanced challenge because ED testimonials can promote ED behaviors. Testimonials in ED interventions must therefore be designed carefully. Some optimal design elements of testimonials are known, but questions remain about testimonial speakers, messaging, and delivery, especially for ED interventions.

**Objective:**

We sought to learn how to design and deliver testimonials focused on positive behavior change strategies within our multisession digital binge eating intervention.

**Methods:**

We applied human-centered design methods to learn users’ preferences for testimonial speakers, messaging, and delivery (modalities, over time, and as “nudges” for selecting positive behavior change strategies they could practice). We recruited target users of our multisession intervention to complete design sessions. Adults (N=22, 64% self-identified as female; 32% as non-Hispanic Black, 41% as non-Hispanic White, and 27% as Hispanic) with recurrent binge eating and obesity completed individual interviews. Data were analyzed using methods from thematic analysis.

**Results:**

Most participants preferred designs with testimonials (vs without) for their motivation and validation of the intervention’s efficacy. A few distrusted testimonials for appearing too “commercial” or personally irrelevant. For speakers, participants preferred sociodemographically tailored testimonials and were willing to report personal data in the intervention to facilitate tailoring. For messaging, some preferred testimonials with “how-to” advice, whereas others preferred “big picture” success stories. For delivery interface, participants were interested in text, video, and multimedia testimonials. For delivery over time, participants preferred testimonials from new speakers to promote engagement. When the intervention allowed users to choose between actions (eg, behavioral strategies), participants preferred testimonials to be available across all actions but said that selectively delivering a testimonial with one action could “nudge” them to select it.

**Conclusions:**

Results indicated that intervention users were interested in testimonials. While participants preferred sociodemographically tailored testimonials, they said different characteristics mattered to them, indicating that interventions should assess users’ most pertinent identities and tailor testimonials accordingly. Likewise, users’ divided preferences for testimonial messaging (ie, “big picture” vs “how-to”) suggest that optimal messaging may differ by user. To improve the credibility of testimonials, which some participants distrusted, interventions could invite current users to submit testimonials for future integration in the intervention. Aligned with nudge theory, our findings indicate testimonials could be used as “nudges” within interventions—a ripe area for further inquiry—though future work should test if delivering a testimonial only with the nudged choice improves its uptake. Further research is needed to validate these design ideas in practice, including evaluating their impact on behavior change toward improving ED behaviors.

## Introduction

Testimonials are an evidence-based behavior change strategy with beneficial impacts on health behaviors. Often characterized by the first-person voice of a typical person [1], testimonials can explain an individual’s experience with a health condition or intervention and can be integrated as part of an intervention’s didactic approach. Testimonial speakers are often credible *sources* (eg, someone who has used an intervention), which is useful because highlighting credible sources is an evidence-based behavior change technique [2]. A meta-analysis by Xu [3] demonstrated that health narratives like testimonials have comparable effectiveness to statistical evidence for supporting behavior change. Specifically, testimonials produce effects on behavioral intentions and are persuasive for health beliefs and attitudes [4]. There is support for using testimonials to encourage health behavior change in a range of health-promoting interventions, including for physical activity [5,6], healthy eating [7], cancer screening [8], smoking cessation [9], and diabetes self-management [10].

In the literature about eating disorders (EDs), the behavioral impacts of testimonials are more nuanced. A small systematic review on mental health recovery narratives identified that in the context of anorexia nervosa, exposure to ED recovery testimonials can increase ED behaviors in testimonial viewers, indicating that particular care should be taken to avoid potentially triggering content in testimonials [11]. Specific to binge eating behaviors, in a qualitative study that exposed young women to video blogs depicting binge eating, participants reported that exposure to testimonials produced both positive (eg, reductions in their own binge eating) and negative effects (eg, desire to replicate depicted binge eating behaviors) [12]. Indeed, personal stories from credible peer sources on so-called “pro-Ana” (anorexia nervosa) and “pro-Mia” (bulimia nervosa) web-based forums can undermine ED recovery and validate ED behaviors [13]. Yet the popularity of these sites among people with EDs [14]—as well as the prominence of online communities [15] and social media influencers [16,17] focused on ED recovery—indicates that people often seek out and are exposed to ED-related testimonials online, such that they may be interested in testimonials from peer sources within interventions as well. To deliver testimonials to users of ED interventions, it is therefore critical to avoid potential safety risks by carefully designing testimonials.

Extant literature provides some guidance for designing safe, effective, and engaging testimonials. De Graaf et al [4] conducted a meta-review of 153 experimental studies of health narratives and identified features of effective testimonials. For testimonial messaging, they found that depicting a positive health behavior in a testimonial (eg, physical activity) is more effective for changing behavioral intentions than depicting the consequences of an unhealthy behavior (eg, health risks associated with a sedentary lifestyle), though this finding was mixed in physical activity studies [18,19]. The review also found that first-person testimonials are most effective, but that users do not find testimonials from speakers with similar characteristics to them (eg, shared gender, race, and age) more persuasive than testimonials from dissimilar speakers. However, others have found that testimonials from speakers with similar characteristics to users are more persuasive for changing health beliefs [20], improving engagement with the health message [21], and increasing behavioral intentions [22]. Indeed, a more recent meta-analysis by Chen et al [23] identified that sharing characteristics with a testimonial speaker improves testimonial persuasiveness. However, to our knowledge, the design of testimonials for ED interventions has not been explored, such that much remains unknown about designing testimonials for populations with EDs.

The purpose of this paper is to inform the design of testimonials for a digital binge eating intervention. This study was conducted as part of our ongoing efforts applying human-centered design methods to create a 16-week digital health intervention (FoodSteps) that targets binge eating and weight-related behaviors. We used human-centered design methodologies because they centralize the perspectives of users [24] and are useful for learning how to carefully design and package psychosocial interventions and their constituent parts [25]. Indeed, including users in human-centered design processes can yield engaging digital tools with high clinical impact [26]. We thus conducted virtual “design sessions” with users to learn their preferences related to testimonials.

We examined several specific components. First, given the behavioral benefits of testimonials and the popularity of ED testimonials in nonintervention contexts (eg, online ED discussion forums) [14,15], we sought to learn if users were interested in viewing testimonials as part of a digital ED intervention. Second, we investigated users’ preferences for testimonial messaging, speakers, and delivery. Within testimonial delivery, we were interested in delivery modalities, as well as learning how to deliver testimonials (1) over time and (2) as “nudges” toward optimal choices within an intervention. Although many digital health interventions that use testimonials are longitudinal (ie, delivered over weeks or months) [27,28], to our knowledge, how to deliver testimonials over time has been unexplored and represents an important design question for motivating ongoing behavior change in a longitudinal intervention. Relatedly, we wanted to learn users’ perspectives on using testimonials to “nudge” a health-oriented behavior, since nudge theory in behavioral economics posits that attaching a “nudge” to an optimal choice is a useful method to influence users’ behavior [29]. Testimonials have been used for nudging in nonhealth contexts [30,31] and could be used in health interventions to nudge users toward a particular choice. We therefore sought to learn whether intervention users are open to testimonials as “nudges” and whether they perceive testimonials delivered as nudges would influence their choices. Collectively, results of this human-centered design study can inform the design of testimonials within health interventions, with particular relevance for interventions that target binge eating and weight-related behaviors.

## Methods

We conducted a qualitative study of FoodSteps target users’ preferences for testimonial design and delivery. In reporting our data, we adhered to the Standards for Reporting Qualitative Research (see supplementary files) [32]. During codebook formation, the research team consisted of 3 bachelor’s level researchers, 1 master’s level researcher, and 3 clinical psychologist researchers.

### Participants and Procedures

From October to December 2022, we recruited potential participants via flyers in Chicago, Illinois, and online via Craigslist, social media, and ResearchMatch. Participants were recruited to participate in design sessions to help design a mobile app for managing binge eating and weight-related behaviors. Through tailored recruitment materials, methods, and locations, we sought to recruit a sociodemographically diverse sample based on gender, race, ethnicity, age, and socioeconomic status in an effort to increase generalizability and relevance.

Interested individuals completed an online screener, and potential participants were invited to complete a baseline assessment to confirm eligibility. Participants were eligible if they were non-pregnant, English-speaking adults (≥18 years old) with regular access to a smartphone. Eligible participants also had obesity (BMI ≥30 kg/m^2^ based on self-reported height and weight) and recurrent binge eating (≥12 objectively large binge eating episodes in the past 3 months based on the diagnostic portion of the Eating Disorder Examination semi-structured interview) [33]. To be eligible, participants had to endorse struggling with losing weight and be willing to use an app to reduce their binge eating. We established this inclusion criteria to obtain a sample of participants that mirrors target users of the FoodSteps intervention. We invited a representative group of 25 participants to our design sessions. Of these, 22 participants completed the interviews; 3 were lost to follow-up.

### Ethical Considerations

This study was approved by Northwestern University’s Institutional Review Board (STU00216998). All participants gave verbal informed consent. Participants received US$25 financial compensation for completing study procedures.

### Design Sessions

The design sessions aimed to learn how to design and deliver testimonials in a digital binge eating intervention. The principal investigator (AKG) and a research assistant (TAM) iteratively developed a 1-hour interview guide for the design sessions. Individual virtual interviews, held via Zoom (Zoom Communications, Inc.), were led by TAM and frequently co-led by AKG.

**Figure 1.**
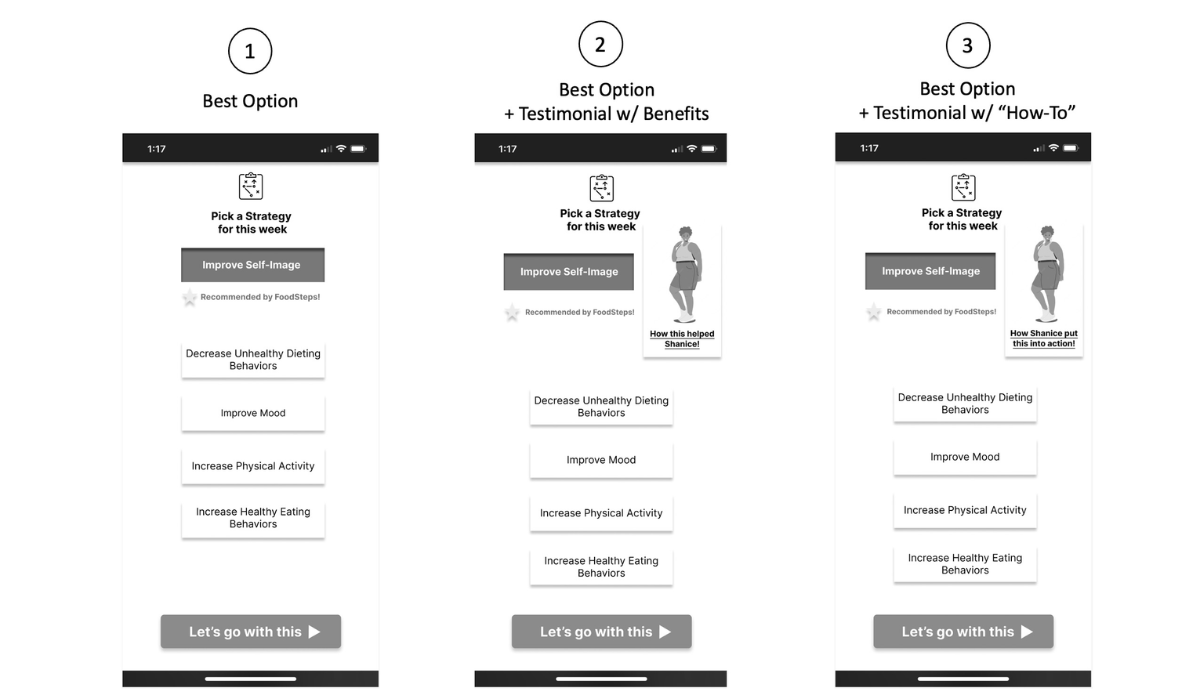
Example slide of testimonial design options shown to participants. Designs iterations include (1) no testimonial, (2) Shanice’s “big picture” testimonial, and (3) Shanice’s “how-to” testimonial. In the latter options, a testimonial is used to nudge a user toward a specific action (in this case, the behavioral strategy “improve self-image”).

During the semistructured design sessions, the research team member leading the interview began by briefly explaining the FoodSteps intervention to the participant. They were then shown nonfunctional mock-ups of intervention features, including designs that featured a testimonial (see Figure 1). Participants were asked which versions of the testimonial designs they preferred and their preferences for testimonial speakers (eg, sociodemographic characteristics), messaging (eg, how-to advice), delivery interface (eg, video), and delivery over time and as a “nudge” to a particular action. Specifically, because FoodSteps involves users choosing behavioral strategies each week, participants were shown designs in which a testimonial is used to nudge users to select a particular strategy.

In Table 1, we present the interview guide questions on testimonials. Because the interviews were semi-structured, we followed-up on participants’ unique responses with additional questions based on the feedback that participants shared.

As saturation emerged (ie, when enough participants provided the same feedback such that the research team felt consensus had been reached), the designs and interview guide were iteratively updated, consistent with the iterative human-centered design process [26]. For example, as saturation developed regarding the set of identity characteristics participants wanted to share with the testimonial speaker, we asked more about participants’ preferences for the longitudinal delivery of testimonials. As the study progressed, we also iteratively updated the visualizations we showed during design sessions based on participants’ feedback. As such, participants were shown slightly different designs, and interviews were focused on different questions depending on the stage of the study at which they completed their design session.

**Table 1.** Guiding questions from our interview guide to learn target users’ preferences for testimonial design and delivery.

#1	What would you want to see in a testimonial?
#2	How would you want that testimonial presented to you (eg, video, article, pictures)?
#3	Is it important to you for the testimonials to be paired with your own identity? (eg, if you are a mother or father, seeing testimonials from other mothers or fathers)
#3a	If yes, ask: In order to make the testimonials related to you, would you be comfortable answering personal questions at the start to inform this personalization?
#4	Would you expect or want to see the same testimonial throughout the program, or different ones?
#5	Which of these designs do you prefer or like best, if any?
#5a	If there is one you prefer, why is it your favorite or what stands out to you about it?
#6	Which design(s) do you like least? What about them do you not like, find confusing, or feel is unhelpful?

### Measures

We assessed participants’ sociodemographic characteristics at screening (gender, race, ethnicity, age, and BMI) and through a questionnaire administered as part of the baseline eligibility assessment (household income and education). Objective binge eating was assessed at baseline by a trained assessor who administered the Eating Disorder Examination interview [33]; this assessor did not conduct the design sessions.

### Analyses

We qualitatively analyzed interview text for themes using methods from thematic analysis [34,35]. Interviews were automatically transcribed by Zoom and edited for accuracy by a research assistant. A research team member (IRR) reviewed the interview data in full, then iteratively generated and applied an initial set of codes using Dedoose (SocioCultural Research Consultants), a qualitative data analysis software. After refining the codebook with another coder (AKG), IRR reviewed and recoded the data and began identifying themes. To strengthen the rigor of the coding process, the full study team reviewed the codebook and discussed the themes. IRR, AO, and AKG refined and named the themes and met often to resolve coding ambiguities.

## Results

### Sample Characteristics

Of the 22 participants, 64% self-identified as female; 27% as Hispanic, 32% as non-Hispanic Black, and 41% as non-Hispanic White. On average, participants were 42 years (range 23-69 years; median 40 years; SD 14.10 years); had a BMI of 39.42 kg/m^2^ (range 30.03-57.56 kg/m^2^; median 37.48 kg/m^2^; SD 7.15 kg/m^2^); and experienced 49 binge episodes over the past 3 months (range 15-111; median 41; SD 30.59). Just over half (54%) had at least a bachelor’s degree. Excluding 4 missing data points, participants’ mean household income was US $79,616 (range US $10,000-$250,000; median US $53,000; SD US $64,991). See Table 2 for full sample characteristics.

**Table 2.** Sample characteristics of the target intervention users who participated in this study’s design sessions.

ID	Gender	Race	Hispanic	Age (y)	Household income (USD)	Education	BMI (kg/m^2^)	Number of binge eating episodes in the past 3 months
1	Female	Black	No	40	—	Master’s degree	44.62	46
2	Female	White	No	36	$205,000	Master’s degree	31.17	41
3	Female	White	No	43	$250,000	Master’s degree	36.94	20
4	Female	White	No	56	$10,000	2-year college	38.16	36
5	Female	White	No	65	—	4-year college	30.99	15
6	Female	Black	No	29	$50,000	High school/GED	46.00	34
7	Female	Black	No	54	$56,000	Master’s degree	47.42	111
8	Female	Black	No	28	—	Some college	57.56	22
9	Female	Black	No	40	$113,104	Professional degree	35.87	64
10	Male	White	No	69	$110,000	Professional degree	36.33	60
11	Female	White	Yes	28	—	4-year college	32.87	18
12	Female	More than one race	Yes	27	$30,000	Some college	35.66	111
13	Male	White	No	68	$60,000	Master’s degree	41.55	84
14	Male	White	Yes	34	$65,000	Master’s degree	34.72	48
15	Female	White	Yes	52	$99,000	Master’s degree	40.85	108
16	Male	White	No	43	$150,000	4-year college	38.01	41
17	Male	White	No	48	$30,000	Some college	54.88	24
18	Female	Black	No	23	$50,000	Some college	38.84	67
19	Male	More than one race	Yes	30	$40,000	Some college	36.59	15
20	Male	Black	No	25	$50,000	Some college	36.18	32
21	Other	White	No	34	$25,000	Some college	42.06	32
22	Female	American Indian or Alaskan Native	Yes	45	$40,000	Some college	30.04	51

### Preferences for Including Testimonials

Most users preferred designs featuring testimonials. They said testimonials personalized the intervention (“*that makes it a little more personalized for you,*” P7) and validated its efficacy (“*look at all these people from various lots of life who have used this and are successful,*” P2). Participants also found testimonials motivating (“*it will encourage someone,*” P6) and engaging:

The testimonial person engages you. It gives you hope. It motivates you.

P19

Many said testimonials fostered connection (“*it’s sort of a camaraderie in a way,*” P13). They said binge eating is an illness for which social support is limited, so testimonials can fill this gap:

It’s good to know that other people have the same issue.

P11

[It] feels more like you’re doing it as a team effort, and that’s […] important with such an isolating illness.

P21

While they generally preferred designs with testimonials, a subset distrusted testimonials for reasons of relevance, authenticity, and utility. A few recommended excluding testimonials altogether. Some participants found testimonials irrelevant because eating and weight are subjective topics:

The reason I don’t like the testimonial option is because it’s a very personal thing that you’re doing.

P3

When it comes to my own weight loss journey, I wouldn’t find how it helped somebody else [useful] […] that’s just not something I would care to look into.

P12

Some found testimonials too “commercial” or distrusted the speaker:

I relate it mentally to too many, not scams, but like trying to sell a product [...] I might think it’s just a paid actor. I don’t trust it enough.

P20

It’s like looking at an infomercial on TV […] Here’s Suzy Cream Cheese, and she lost four hundred pounds in one week.

P10

A few participants also said that they would prefer advice come from the intervention, not a former user:

The ‘how-to’ is more important to me than the testimonial.

P16

Having some instruction as to how I can put it into action might be more beneficial for me personally than hearing how someone else put it into action.

P9

Participants also cited unique reasons for disliking testimonials. This participant said the speaker’s success did not guarantee their own, so an unhelpful self-comparison could ensue:

If it doesn’t help you and it did help someone else, then how are you going to internalize that? That’s why I didn’t like that one.

P3

Other critiques were of the illustration’s larger body size, which raised concerns about the intervention’s efficacy (“*if this helps Shanice, why does Shanice still look like this?*”*,* P9) and prompted size comparisons (“*am I fatter than this person?*”, P15). One participant worried the speaker’s racial identity could alienate users (“*I would not use the image and the name, just because our country is so* [...] *ugly about race, and that could turn some people off,*” P4).

### Design Preferences

Participants described their preferences for testimonial speakers, interface, and messaging.

#### Speaker

Participants preferred speakers with shared identity characteristics such as age, race, gender, body size, lifestyle (ie, being busy), parenthood, and socioeconomic status.

I would immediately look at it and say I’m not a middle-aged African American woman.

P10

If it was someone smaller, I would be a bit more skeptical. But if it was someone that looks like me, I could say okay, she or he goes through the same struggle as I do.

P18

She’s a busy lady like you are, and […] she still found time to squeeze it in.

P1

There’s different challenges if you’re a working mother compared to a stay-at-home mother, or someone who’s not a mother.

P2

When Oprah lost her weight it was like, you have a personal chef, it’s easy for you.

P7

Participants said sharing characteristics with speakers made those testimonials more comfortable (“*it’s just something I feel comfortable with,”* P22), relevant (“*I don’t want to see how somebody was successful who I can’t relate to,*” P10), and inspiring (“*if it’s someone I can relate to* […] *it makes it more real,*” P7). They said these testimonials would make them more likely to listen:

If it shows some 6-foot tall, muscular dude […] I’m not going to listen to him.

P8

Yet, one user cautioned that sharing characteristics does not assure identification (“*just because this person is a female, and I’m a female, does not mean that I identify with this person,*” P1).

Understanding which intersecting identities matter most to which users is important. We asked a subset of participants if they would be willing to report personal data in the intervention to facilitate identity-based matching and personalization; all of them said yes.

When you introduce that [the demographic questions] you should say: in order to really focus in on you, we’re going to ask you these questions […] I want to know you’re focusing in on me, because then it’s worth my while to continue.

P10

These findings indicate that it will be important for interventions to deliver tailored testimonials to users, which can be facilitated by assessing their identity characteristics within the intervention.

#### Messaging

Generally, participants were shown 2 types of testimonials (see Figure 1). The first (“how this helped Shanice”) conveyed the “big picture” impact of the behavioral strategy; the second (“how Shanice put this into action”) conveyed “how-to” advice. Participants reported varying preferences between these options. Those who preferred “big picture” testimonials found the speaker’s end results more motivating than the speaker’s process (“*I’m more excited about the final results of losing weight, so this is more engaging*,” P19). Others thought the big picture impact was obvious (“*it’s not rocket science […] they know the benefits*,” P8).

Some participants found “how-to” advice unhelpful (“*how one put it into action may not be how someone else puts it into action,*” P9). Others favored “how-to” testimonials for their actionable insights (“*it’s concrete. Easily implementable,*” P14) that operationalize behavior change:

Sometimes when things are broken down […] it makes it a lot easier, especially if it’s something that’s new to you.

P18

These varying preferences indicate that both types of testimonial messaging (“big picture” and “how-to”) are motivating to different users. To that end, this participant recommended assessing users’ preferences within the intervention to discern the optimal messaging to motivate a user:

I could see there being an option in the setup for the app to choose […] Do you want to know testimonials? Do you want how-to? Which ones do you want?

P17

Understanding what testimonial messaging to deliver and to whom to best facilitate motivation is critical to optimize testimonials’ behavioral impact.

#### Interface

Participants wanted testimonials with a simple interface and varying multimedia, such as videos, to encourage engagement and connection (“*it’s more a personal connection when you have a video,*” P2). A participant said they would prefer an image of the speaker, instead of an illustration (“*to the extent it could be a real person, that would be helpful,*” P9). Others preferred testimonials delivered multimodally (eg, text and video) to give options for viewing the content:

Maybe having a video, and then the transcript below […] so I can kind of listen in my head where I may not have the audio, but I can still read and see them speaking.

P2

Every week, if there’s something different; sometimes there might be audio of somebody talking about their experience, then there might be a write-up, and then there might be a video […] that might be motivating […] something that changes as weeks go by.

P1

One participant wanted multiple testimonials available so they could choose between speakers:

If you had a choice, like if you wanted to see Shanice, or Bob, or whomever. Like you could watch multiple testimonials from different people.

P2

Taken together, these preferences for multimodal content and choice between speakers can inform how intervention designers optimize testimonials’ user interface.

### Delivery Preferences

Testimonials could be a strategy to nudge users toward a particular action within the intervention when choice is involved (eg, in our intervention, users choose between behavioral strategies). We therefore asked participants about their preferences for seeing testimonials when there is an element of user choice. If multiple actions are available, this participant said a testimonial should only be delivered with the nudged action:

If I clicked on ‘mood’ this week, because ‘mood’ is not my recommended, there would be no testimonial […] I think I would be more motivated [to select the recommendation] if there was a testimonial.

P2

Other participants preferred to see testimonials across all available actions:

I thought it was going to show a visual next to [each behavioral strategy]. Like, on ‘physical activity,’ I thought it would have been somebody with weights, and on ‘behaviors,’ it would have somebody eating.

P8)

It would be great if even if it wasn’t a recommendation, that they would have a testimonial there, too […] I still would like that option added to it.

P7)

Additionally, because most behavioral interventions are multi-session, we sought to understand users’ preferences for the longitudinal delivery of testimonials. In our design sessions, we assessed this by asking users about their preferences after envisioning a week had passed in the program. Many participants wanted testimonial speakers to vary over time because new testimonials continue validating the intervention’s efficacy:

It validates [it] if you can see real life people and every day it’s a brand-new person.

P19)

I think different people each week. So that way you can see a variety of people, so you don’t get into, like, oh my gosh, only one person’s used this.

P2)

Some preferred testimonials from varied speakers each week to reduce potential boredom from hearing the same person’s story repeatedly (“*especially if they’re going through this week after week […] I really don’t need to see that again,*” P17). Delivering varied testimonials over time also increases the odds of identifying with the speaker:

If I click on it one day, and I see a Tom or something […] I don’t really connect if it were Tom, but maybe next week I get somebody else.

P1)

However, one participant wanted to see the same speaker over time (“*I’d feel a lot more impressed if you showed me Bob all the time […] It’s just like having a personal trainer,*” P10). These findings indicate that when choice is involved, selectively delivering testimonials could nudge users toward the optimal action, though users preferred testimonials be available across all actions. Understanding how to deliver testimonials over time will be crucial for intervention designers to facilitate motivation and engagement throughout a multi-session intervention.

## Discussion

### Principal Findings

We conducted design sessions to learn how to design and deliver testimonials within a multisession digital binge eating intervention. We found that participants generally preferred designs with testimonials because they promoted engagement, motivation, and connection. This finding indicates that target users of a digital intervention for binge eating and weight-related behaviors are interested in viewing testimonials as part of the intervention and aligns with other literature indicating interest in testimonials from peer sources among individuals with EDs [14,15]. We also described participants’ preferences for testimonial design and delivery over time. From these results, we collated design recommendations, summarized in Table 3.

**Table 3.** Recommendations for optimizing the design of testimonials in digital health interventions.

General	Include testimonials from former users as part of digital interventions for eating and weight-related behaviors.
	Assess users’ testimonial preferences in-app and incorporate choice around whether and how to receive testimonials (eg, speaker, messaging, or delivery).
Speaker	Design testimonials that match the user sociodemographically to improve relevance, understanding, and listening.
	Highlight the authenticity of the speaker to establish trust and avoid appearing too “commercial”.
	Avoid showing users irrelevant testimonials that can disengage them.
	Facilitate sociodemographic matching of testimonials by leveraging users’ willingness to input personal information in the app for matching purposes.
	Recognize that some users may prefer following the same person over time; as feasible, create and disseminate this content.
Messaging	Highlight actionable, nonprescriptive content that recognizes the personal nature of health journeys to transfer information without alienating users. eg,*“See how Shanice made this strategy work for her. Her unique approach might motivate you to find your own way to increase your daily activity.”*
	Highlight the “big picture” impact of behavior changes to inspire users. eg,*“Here’s how increasing her daily physical activity made Shanice feel stronger and helped her connect with her kids.”*
	Include language to help users avoid fear of failure or unproductive self-comparison to the testimonial speaker. eg,*“The key is finding an approach that fit in my life. For me, finding what worked was a personal journey, and every small step has added up to big changes.”*
Interface	Deliver testimonials through images, video, and audio to foster connection between the viewer and speaker, and capture motivating effects.
	Vary delivery method of testimonials to engage users.
Delivery	Offer testimonials from new speakers weekly (or on an intervention-appropriate cadence).
	When choice between actions is involved, consider leveraging testimonials to nudge users toward the optimal action.

Participants preferred testimonial speakers who shared their sociodemographic characteristics. This finding contrasts with de Graaf’s [4] meta-analysis, but aligns with more recent research affirming the effectiveness of culturally tailored testimonials [36] and the persuasive influence of sharing race, age, and gender with a testimonial speaker [37]. When asked why tailored testimonials mattered to them, some participants said that nonrelatable testimonials (eg, from speakers with higher socioeconomic status or different racial identities) could alienate users, which underscores the importance of tailoring testimonials to users’ personal characteristics. Notably, tailoring based on body size poses a nuanced challenge for weight-related interventions. While most participants preferred speakers with relatable body sizes, one said that this would make them think the intervention was ineffective. Indeed, because sharing certain characteristics was important to some participants, but not others, our results indicate that intervention designers should consider ways to assess users’ preferences and characteristics at the onset of the intervention to identify users’ most pertinent identities (eg, is it more important for testimonials to match a user’s gender or socioeconomic status?) and tailor testimonials accordingly. This can be facilitated by users’ willingness to report personal information in the intervention, though continued design work is needed to understand user tolerance for testimonial personalization over time. Indeed, Robbins et al [38] observed that some Black participants were uncomfortable with a video testimonial intervention that featured overwhelmingly Black speakers. In multisession interventions, testimonial tailoring based on characteristics like race could feel increasingly targeted over time. It is thus necessary to learn users’ preferences for tailored testimonials throughout an intervention during its actual delivery. Further research is also needed to empirically test if tailoring testimonials improves their effectiveness in the context of eating and weight-related interventions.

For testimonial messaging, we found users were relatively split between receiving “how-to” information versus “big picture” information, in that some preferred testimonials with actionable advice, whereas others preferred testimonials that highlighted the end result of the intervention. This divide also appears in the literature on testimonials for deterring maladaptive health behaviors. While Keer et al [39] found that testimonials describing the positive consequences of abstinence from binge drinking were most effective, Jawed and Hogan [40] found that highlighting actionable strategies for smoking cessation improved testimonial effectiveness and engagement. Our findings affirm that both forms of testimonial messaging may be effective for different subsets of users, depending on their preferred style of messaging. To this end, participants suggested incorporating choice around whether and how to receive testimonials, depending on the elements they find most motivating. User-driven personalization is highly feasible in digital interventions, given a sufficient bank of testimonial content, such that this is a promising area for further inquiry. Future work also would benefit from identifying factors that predict which design elements (eg, forms of messaging) are preferred by which subsets of users, toward effectively personalizing testimonials to users. Importantly, we did not explore users’ preferences for testimonial messaging that included descriptions of binge eating or dangerous compensatory behaviors, given the behavioral consequences that can arise when viewers of ED testimonials are exposed to this kind of messaging [11,12]. Future work could explore opportunities to safely design testimonials that mention such behaviors, though our results indicate that potential users of our binge eating intervention were interested in and motivated by testimonials about positive health behaviors, such that testimonials describing ED behaviors could be avoided in interventions to reduce potential risks.

For delivery, we found that users were interested in written testimonials as well as video, audio, and multimedia testimonials. Because de Graaf [4] did not identify any modality as uniquely effective, and given participants’ varied preferences, assessing users’ preferred delivery modality at the onset of intervention is another promising opportunity for tailoring. For delivery over time, users preferred testimonials from varied speakers because they were more engaging and validated the intervention’s efficacy. We did not learn how their messaging preferences may change over time, which is important because these preferences could meaningfully evolve (eg, a new user initially prefers “big picture” testimonials but later prefers “how tos”). Future work should thus examine methods to longitudinally assess users’ testimonial preferences. When the intervention allowed users to choose between actions (in our case, behavioral strategies), users preferred testimonials to be delivered across all choices. While intervention designers could do this, our results indicate that selectively delivering a testimonial with the recommended action could encourage users to select it. This finding aligns with nudge theory in behavioral economics [29] and may be especially relevant for just-in-time adaptive interventions, which have deployed nudges as part of their behavior change strategies [41]. Our finding that users positively perceived testimonials indicates that testimonials are a promising design element to explore for deployment as nudges in digital health interventions, though future work should test if exclusively delivering a testimonial for the optimal action improves the action’s uptake.

Despite the generally positive perceptions of testimonials, some users said testimonials would not motivate behavior change. This finding needs to be validated in practice through actual intervention delivery. Although much research supports the behavioral effects of testimonials [3], including for promoting abstinence from maladaptive health behaviors [9], Shen et al [42] found that cessation testimonials did not have significant effects, as opposed to prevention testimonials (eg, using condoms) and detection testimonials (eg, cancer screening), which did produce significant effects. This indicates that intervention designers—especially those who are designing interventions to dissuade maladaptive health behaviors—must continue identifying other elements to influence behavior and design to preempt testimonial pitfalls. Indeed, the subset of users who said testimonials would not influence their health behavior change universally critiqued the testimonials’ trustworthiness. This objection can be understood through the lens of persuasive intent (ie, a message’s implicit goal to persuade), which has been shown to undermine the credibility and persuasiveness of health communications [43,44]. Designing testimonials to highlight source credibility may help improve trust in the testimonial message [45]. Another approach to improve the credibility of testimonials could involve inviting users to submit their own testimonials for future integration into the intervention. This strategy could also help create a bank of testimonials from a diversity of users, which could then be deployed in the intervention.

Further research is needed to validate these findings with FoodSteps users in practice. While we believe these findings are relevant to interventions for other health topics, our study is limited by its focus on an intervention for binge eating and weight-related behaviors. Designers of other health interventions may wish to follow similar human-centered design methods to identify intervention-specific design preferences.

### Conclusions

This study sought to learn how to optimize the design and delivery of testimonials in a multisession digital health intervention. Our findings confirm that target users of a digital intervention for binge eating and weight-related behaviors wanted testimonials delivered as part of the intervention. We generated novel insights into users’ preferences for testimonial messaging, speakers, and delivery (through varied modalities, over time, and as a “nudge” toward a particular action). These results indicate opportunities for digital health intervention designers to tailor testimonials to users based on their preferences and identities. This study extends existing research by applying human-centered design methods to identify design preferences that can now be implemented and tested in practice. Our work offers a foundation for further inquiry into areas such as user preferences for sociodemographic tailoring over time and testimonials as “nudges” toward optimal actions in health interventions. Future research should continue applying human-centered design methods to address these and other questions, toward the goal of optimizing the behavioral impact of health testimonials.

## Supplementary material

10.2196/59691 Checklist 1 SRQR checklist.

## Abbreviations

ED: eating disorder

## References

[1] Bilandzic H, Busselle R (2013). The SAGE Handbook of Persuasion: Developments in Theory and Practice.

[2] Michie S, Richardson M, Johnston M (2013). The behavior change technique taxonomy (v1) of 93 hierarchically clustered techniques: building an international consensus for the reporting of behavior change interventions. Ann Behav Med.

[3] Xu J (2023). A meta-analysis comparing the effectiveness of narrative vs. statistical evidence: health vs. non-health contexts. Health Commun.

[4] de Graaf A, Sanders J, Hoeken H (2016). Characteristics of narrative interventions and health effects: a review of the content, form, and context of narratives in health-related narrative persuasion research. RCR.

[5] Perrier MJ, Martin Ginis KA (2018). Changing health-promoting behaviours through narrative interventions: a systematic review. J Health Psychol.

[6] Falzon C, Radel R, Cantor A, d’Arripe-Longueville F (2015). Understanding narrative effects in physical activity promotion: the influence of breast cancer survivor testimony on exercise beliefs, self-efficacy, and intention in breast cancer patients. Support Care Cancer.

[7] Oh HJ, Larose R (2015). Tell me a story about healthy snacking and I will follow: comparing the effectiveness of self-generated versus message-aided implementation intentions on promoting healthy snacking habits among college students. Health Commun.

[8] Perrier MJ, Martin Ginis KA (2017). Narrative interventions for health screening behaviours: a systematic review. J Health Psychol.

[9] Igartua JJ, Rodríguez-Contreras L (2020). Narrative voice matters! Improving smoking prevention with testimonial messages through identification and cognitive processes. Int J Environ Res Public Health.

[10] Gardner L, Leshner G (2016). The role of narrative and other-referencing in attenuating psychological reactance to diabetes self-care messages. Health Commun.

[11] Rennick-Egglestone S, Morgan K, Llewellyn-Beardsley J (2019). Mental health recovery narratives and their impact on recipients: systematic review and narrative synthesis. Can J Psychiatry.

[12] Sung B, Lee J yeon (2024). The influence of YouTube binge eating vlog viewing on perception and behavior among South Korean women, aged 20s to 30s, with binge eating tendencies: a qualitative analysis. Int J Ment Health.

[13] Firkins A, Twist J, Solomons W, Keville S (2019). Cutting ties with Pro-Ana: a narrative inquiry concerning the experiences of Pro-Ana disengagement from six former site users. Qual Health Res.

[14] Sharpe H, Musiat P, Knapton O, Schmidt U (2011). Pro‐eating disorder websites: facts, fictions and fixes. J Public Ment Health.

[15] Nutley SK, Falise AM, Henderson R, Apostolou V, Mathews CA, Striley CW (2021). Impact of the COVID-19 pandemic on disordered eating behavior: qualitative analysis of social media posts. JMIR Ment Health.

[16] Nikolova I, LaMarre A (2023). “If I Unfollow Them, It’s Not A Dig at Them”: A narrative analysis of Instagram use in eating disorder recovery. Psychol Women Q.

[17] Herrick SSC, Hallward L, Duncan LR (2021). “This is just how I cope”: an inductive thematic analysis of eating disorder recovery content created and shared on TikTok using #EDrecovery. Int J Eat Disord.

[18] Gray JB, Harrington NG (2011). Narrative and framing: a test of an integrated message strategy in the exercise context. J Health Commun.

[19] Wirtz JG, Kulpavaropas S (2014). The effects of narrative and message framing on engagement and eating intention among a sample of adult Hispanics. J Nutr Educ Behav.

[20] de Graaf A (2014). The effectiveness of adaptation of the protagonist in narrative impact: similarity influences health beliefs through self-referencing. Hum Commun Res.

[21] Kim M, Shi R, Cappella JN (2016). Effect of character-audience similarity on the perceived effectiveness of antismoking PSAs via engagement. Health Commun.

[22] Lu AS (2013). An experimental test of the persuasive effect of source similarity in narrative and nonnarrative health blogs. J Med Internet Res.

[23] Chen M, Dong Y, Wang J (2024). A meta-analysis examining the role of character-recipient similarity in narrative persuasion. Communic Res.

[24] Maguire M (2001). Methods to support human-centred design. Int J Hum Comput Stud.

[25] Lyon AR, Koerner K (2016). User-centered design for psychosocial intervention development and implementation. Clin Psychol (New York).

[26] Graham AK, Wildes JE, Reddy M, Munson SA, Barr Taylor C, Mohr DC (2019). User-centered design for technology-enabled services for eating disorders. Int J Eat Disord.

[27] Brewer LC, Kumbamu A, Smith C (2020). A cardiovascular health and wellness mobile health intervention among Church-going African Americans: formative evaluation of the FAITH! app. JMIR Form Res.

[28] Bruehlman-Senecal E, Hook CJ, Pfeifer JH (2020). Smartphone app to address loneliness among college students: pilot randomized controlled trial. JMIR Ment Health.

[29] Patel MS, Volpp KG, Asch DA (2018). Nudge units to improve the delivery of health care. N Engl J Med.

[30] Gottschewski-Meyer PO, Lüddemann N, der LM, von VC (2023). Exploring digital social norms nudges in E-grocery: typical consumer testimonials with a warm glow. https://aisel.aisnet.org/wi2023/59.

[31] Ouvrard B, Préget R, Reynaud A, Tuffery L (2023). Nudging and subsidising farmers to foster smart water meter adoption. Eur Rev Agric Econ.

[32] O’Brien BC, Harris IB, Beckman TJ, Reed DA, Cook DA (2014). Standards for reporting qualitative research: a synthesis of recommendations. Acad Med J Assoc Am Med Coll.

[33] Fairburn CG, Cooper Z, O’Connor ME (2014). The Eating Disorder Examination (EDE) 17.0D.

[34] Braun V, Clarke V (2006). Using thematic analysis in psychology. Qual Res Psychol.

[35] Braun V, Clarke V (2021). Can I use TA? Should I use TA? Should I *not* use TA? Comparing reflexive thematic analysis and other pattern‐based qualitative analytic approaches. Couns Psychother Res.

[36] Nugraha YR, Aditjondro E, Svenson D (2024). Harnessing the power of testimonial videos: a deep dive into the #1Minute2Quit campaign for spreading awareness on the health hazards of tobacco. JKMJC.

[37] Kim M (2019). When similarity strikes back: conditional persuasive effects of character-audience similarity in anti-smoking campaign. Hum Commun Res.

[38] Robbins R, Senathirajah Y, Williams NJ (2019). Developing a tailored website for promoting awareness about obstructive sleep apnea (OSA) among Blacks in community-based settings. Health Commun.

[39] Keer M, van den Putte B, de Wit J, Neijens P (2013). The effects of integrating instrumental and affective arguments in rhetorical and testimonial health messages. J Health Commun.

[40] Jawed A, Hogan A (2024). Lessons learned from telltale testimonies: a descriptive study assessing coverage of the tips from former smokers campaign on YouTube. Am J Health Educ.

[41] Hsu TCC, Whelan P, Gandrup J, Armitage CJ, Cordingley L, McBeth J (2025). Personalized interventions for behaviour change: a scoping review of just-in-time adaptive interventions. Br J Health Psychol.

[42] Shen F, Sheer VC, Li R (2015). Impact of narratives on persuasion in health communication: a meta-analysis. J Advert.

[43] Lee EJ, Kim HS, Joo MH (2023). Social media vs. mass media: mitigating the suspicion of ulterior motives in public health communication. Health Commun.

[44] Wang W, Shen F (2019). The effects of health narratives: examining the moderating role of persuasive intent. Health Mark Q.

[45] Xu Y, Margolin D, Niederdeppe J (2021). Testing strategies to increase source credibility through strategic message design in the context of vaccination and vaccine hesitancy. Health Commun.

